# Renal Involvement of CD20-Negative Intravascular Large B Cell Lymphoma with Neurological Manifestations

**DOI:** 10.1155/2022/8613965

**Published:** 2022-09-10

**Authors:** Faten Aqeel, Serena M. Bagnasco, Duvuru Geetha

**Affiliations:** ^1^Division of Nephrology, Johns Hopkins Hospital, Baltimore, MD, USA; ^2^Department of Pathology, Johns Hopkins Hospital, Baltimore, MD, USA

## Abstract

The involvement of hematological tumors such as lymphoma in the kidneys is a well-recognized phenomenon. Some of the distinct reported pathological processes resulting in kidney dysfunction include minimal change disease, lymphocytic invasion of the parenchyma, immune complex disposition, immunotactoid glomerulopathy, membranous glomerulopathy, and acute tubular injury. We report a rare case of CD20-negative intravascular lymphoma found on a kidney biopsy in a male with primary angiitis of the central nervous system (CNS) who presented with acute kidney injury and proteinuria. After the initiation of rituximab, cyclophosphamide, doxorubicin hydrochloride, vincristine sulfate, and prednisone (R-CHOP), kidney function improved and proteinuria resolved.

## 1. Introduction

Intravascular large B cell lymphoma (IVLBCL) is a rare subtype of extranodal non-Hodgkin lymphoma. It is characterized by the tendency of malignant cells to proliferate in the intraluminal space of small and medium vessels, sparing surrounding tissues [1]. The incidence rate of IVLBCL is 0.095 cases per 1,000,000 in the United States, with increasing incidence in recent years due to increased recognition [2]. The median age of diagnosis is between 60 and 70 years old with no clear sex preference [3]. The nervous system and skin have the highest incidence of involvement [4]. Renal involvement is rare, with only case reports published in the literature describing its manifestation.

We describe a patient with primary angiitis of the central nervous system, on treatment with intravenous rituximab and corticosteroids, who presented with acute kidney injury and proteinuria. Kidney biopsy showed findings consistent with IVLBCL. Improvement in serum creatinine and proteinuria ensued the following therapy with R-CHOP.

## 2. Case Report

A 59-year-old man presented with acute kidney injury and proteinuria. He has a history of primary angiitis of the central nervous system (CNS) diagnosed one year before his presentation. His primary angiitis of the CNS initially manifested with recurrent admissions for various neurological deficits, including vertigo, gait instability, expressive aphasia, and cranial nerve palsies. Magnetic resonance imaging (MRI) of the brain showed multiple subacute ischemic strokes. A formal 4-vessel cerebral angiogram showed several fusiform aneurysmal dilations of cerebral arteries in the posterior fossa concerning for vasculitis. Brain biopsy revealed nonspecific focal leptomeningeal acute inflammation. Immunostaining for beta-amyloid highlighted scattered neocortical deposition and was negative in the vascular walls. No other stains were done. A lumbar puncture revealed a high total protein of 122 mg/dL (reference value 15–45 mg/dL) but was otherwise unremarkable. Serological studies were negative, including antineutrophilic cytoplasmic autoantibodies (ANCA) and antinuclear antibodies (ANA). The diagnosis of primary angiitis of the CNS was made based on his imaging findings, and treatment with intravenous rituximab and corticosteroids ensued. He received intravenous rituximab (two doses of 1 g, two weeks apart) in addition to intravenous pulse methylprednisolone 1 g for 5 days with improvement in his cognition. This was followed by 100 mg oral prednisone daily for 1.5 months and a slow taper. Four months later, he presented with worsening neurological symptoms while on 20 mg prednisone daily. Rituximab was administered again (two doses of 1 g, two weeks apart) with significant improvement. At six months and one year of treatment initiation, he remained on 5 mg prednisone daily with no recurrence of neurological symptoms.

One year into his diagnosis of primary angiitis of the CNS, routine laboratory testing showed a serum creatinine level 1.57 mg/dL from a normal baseline (reference value 0.4–1.2 mg/dL), blood urea nitrogen 18 mg/dL (reference value 7–25 mg/dL), serum albumin 4.2 g/dL (reference value 3.6–5.1 g/dL), hemoglobin 10.3 g/dL (reference value 13.9–16.3 g/dL), lactate dehydrogenase 351 U/L (reference value 100–190 U/L), haptoglobin 90 mg/dL (reference value 43–212 mg/dL), and normal complement levels (C3 and C4). Urinalysis showed 2^+^ proteinuria without any hematuria or pyuria. Urine protein-to-creatinine ratio (UPCR) was 500 mg/g. Serological workup including serum protein electrophoresis (SPEP), immunofixation, and kappa/lambda ratio was negative for monoclonal gammopathy. Antineutrophilic cytoplasmic autoantibody (ANCA) was negative. Renal ultrasound showed the right kidney measuring 11.1 cm and the left kidney measuring 12.0 cm with normal echogenicity. There was no evidence of masses, hydronephrosis, or stones.

**Figure 1:**
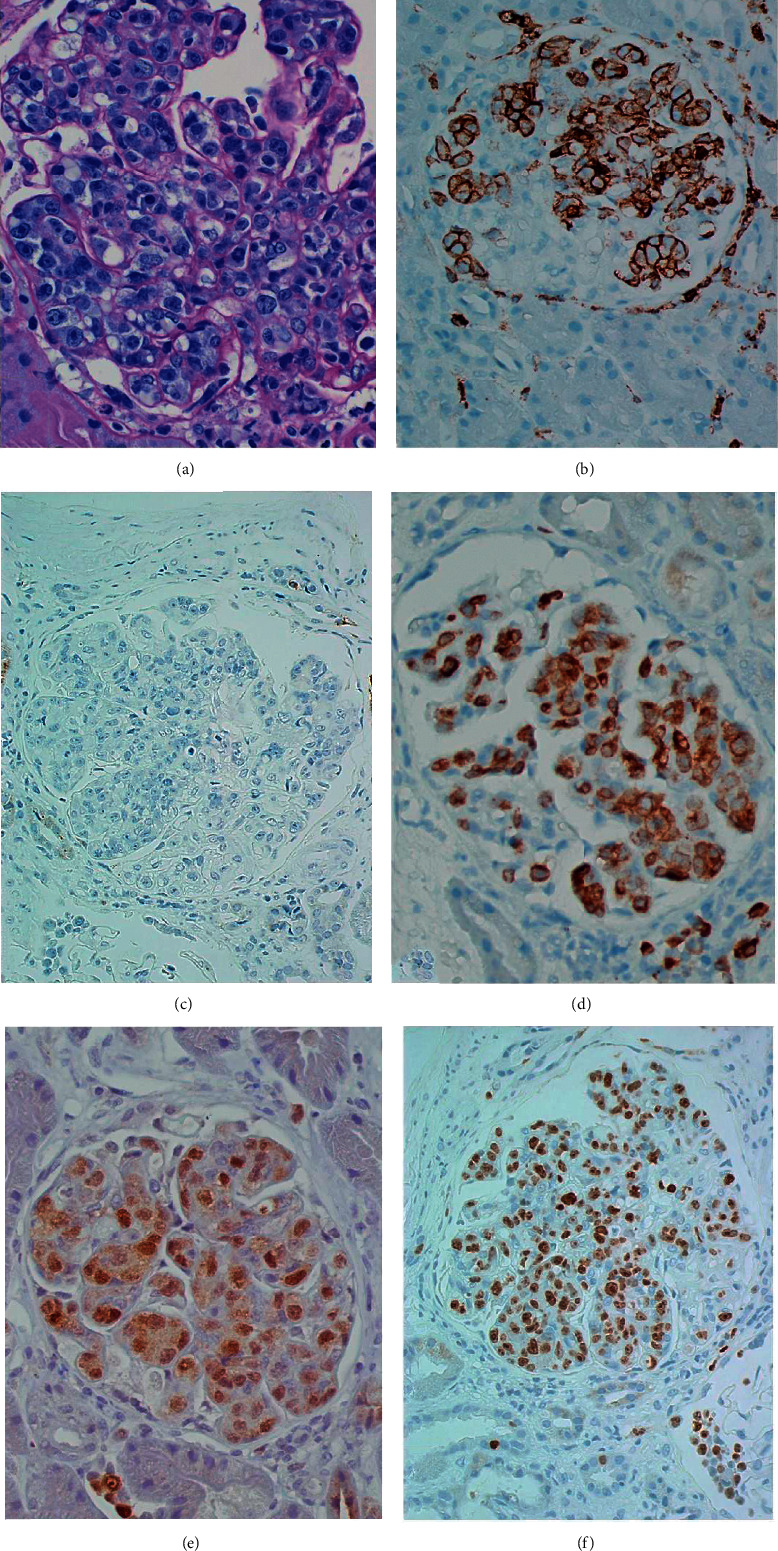
Intraglomerular lymphoma cells. (a) PAS large atypical cells obstructing the glomerular capillary lumen (magnification 600x). (b) Intracapillary glomerular cells positive for CD45 (magnification 400x). (c) Intracapillary glomerular cells negative for CD20 (magnification 400x). (d) Intracapillary glomerular cells positive for CD79A (magnification 400x). (e) Intracapillary glomerular cells positive for MUM-1 (magnification 400x). (f) Intracapillary glomerular cells highly positive for KI-67 (magnification 400x).

A percutaneous kidney biopsy was performed. Light microscopy showed nine glomeruli, two partially sclerosed with mild tubulointerstitial scarring. Several glomeruli appeared enlarged and hypercellular (Figure 1). The glomerular capillary lumen showed atypical cells with large nuclei, prominent nucleoli, and rare mitotic figures. Occasionally, these atypical cells were noted within the tubular epithelial walls and in rare peritubular capillaries. The atypical cells showed positive staining for CD45, Mum-1, CD79a, and KI-67 and negative staining for CD3, CD5, CD10, CD20, CD30, CD56, CD138, ALK, BCl-6, Pax-5, and HHV8. No immune deposits were detected by immunofluorescence and electron microscopy.

Based on the immunophenotype of CD10-negative, BCl-6-negative, and MUM-1-positive and the diagnosis of intravascular large B cell lymphoma, nongerminal center B cell subtype was made per the Hans classification [5].

**Figure 2:**
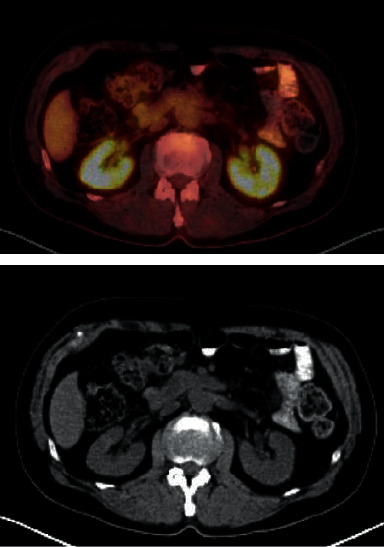
Diffuse symmetric fluorodeoxyglucose (FDG) uptake within the bilateral renal parenchyma on positron emission tomography-computed tomography (PET-CT) scan.

A referral to hematology/oncology was made. Positron emission tomography-computed tomography (PET-CT) scan revealed increased fluorodeoxyglucose (FDG) uptake in the kidneys bilaterally (Figure 2), left C2 and right C4, C5 concerning the perineural invasion of nerve roots. There was no evidence of skin rash, but a skin biopsy was done which did not show evidence of lymphoma. Intravascular large B cell lymphoma with renal and central nervous system involvement was diagnosed. Serum creatinine level and proteinuria peaked at 2.2 mg/dL and 700 mg/g, respectively, before the initiation of chemotherapy. His Eastern Cooperative Oncology Group (ECOG) performance status was 2 at that time. After three cycles of R-CHOP and intrathecal chemotherapy over two months, serum creatinine improved to 1.3 mg/dL with complete resolution of proteinuria and improvement in neurological symptoms. After six cycles of R-CHOP and intrathecal chemotherapy over four months, serum Cr was 1.1 mg/dL with no proteinuria. Unfortunately, new neurological deficits were noted with widespread cranial nerve involvement on PET-CT concerning the relapsed disease. He was started on high-dose methotrexate plus rituximab and ultimately received chimeric antigen receptor therapy (CAR-T). Unfortunately, his clinical status deteriorated, and he expired three months later.

## 3. Discussion

The diagnosis of IVLBCL has been increasingly recognized in recent years. Despite that, it remains a diagnostic challenge given the variability in clinical presentations and the delay in diagnosis without a tissue biopsy. Previously, two clinical variants were widely accepted: A Western variant, characterized by the CNS and skin involvement; and an Asian variant, characterized by fevers, multiorgan involvement, and hepatosplenomegaly [6]. Most recently, three different variant types, classical (characterized by organ-involved symptoms), cutaneous, and hemophagocytic syndrome-associated, have been described given differences in outcomes [7]. The most common presentation is fever (90%), cytopenias (90%), and confusion (50%) [8]. IVLBCL involving the kidney directly has been only described in case reports. A review of 43 published case reports concluded that the most common renal manifestation is proteinuria (90.0%), followed by renal failure (60.5%) and nephrotic syndrome (21.0%) [9]. Despite significant improvement in outcomes with chimeric anti-CD20 monoclonal antibody (rituximab-based) chemotherapy, relapses are still common.

In our patient, the findings in light microscopy and the immunophenotypic pattern met the diagnostic criteria for IVLBCL. Compared to the case reports published in the literature, this case is unusual given the lack of significant symptom burden on presentation and negative CD20 cells. At the time of the kidney biopsy, our patient was treated with intravenous rituximab for his primary angiitis of the CNS, which likely eliminated CD20-positive cells, leading to a unique presentation.

Quintero Vega et al. [10] described a case of a patient who initially presented with fevers, dyspnea, arthralgia, and headaches and was found to have anemia and an elevated level of lactate dehydrogenase. A bone marrow biopsy was negative for neoplasm or hemophagocytic syndrome. Rapid onset of nephrotic syndrome with an unclear degree of proteinuria ensued and warranted a kidney biopsy, which showed atypical lymphocytes located in the glomerulus and tubules, making the diagnosis of IVLBCL. Another case of IVLBCL confirmed with a kidney biopsy had a similar presentation with fevers and dyspnea followed by rapid onset of nephrotic syndrome with UPCR 4.94 g/g and normal kidney function with negative bone marrow biopsy results [9]. Similarly, another case proved the diagnosis of IVLBCL with a kidney biopsy but negative bone marrow biopsy results [11]. This raises the question of the utility of bone marrow biopsies when there is evidence of other organ involvement.

Histologically, atypical cells usually express B cell markers such as CD20, CD79a, BCl-2, BCl-6, and MUM-1 and variable expressions of CD50 and CD10. Of the 43 cases of IVLBCL with renal involvement reviewed by Kim et al. [9], 35 patients exhibited infiltration of the malignant cells within the glomerular capillaries, with or without peritubular and interstitial involvement. The tumor cells were localized in peritubular capillaries without glomerular involvement in four cases. The remaining three cases received a presumptive diagnosis of intrarenal IVLBCL given kidney dysfunction ± bilateral kidney enlargement on imaging, with a tissue diagnosis of IVLBC made by means other than a kidney biopsy [9].

In terms of imaging, enlargement of the kidneys on radiologic evaluation can be seen in 34.3% of the cases [9]. In our case, a PET-CT scan revealed increased (FDG) uptake in the kidneys bilaterally without marked enlargement and swelling, as seen in some other case reports [12, 13]. In a case series of ten patients, a skin biopsy was performed on three patients with cutaneous lesions, making the diagnosis of IVLBCL. For those who did not have skin lesions or cytopenias, the diagnosis of IVLBCL was made by bone marrow biopsies after increased FDG uptake was noted on PET/CT. Therefore, PET/CT may be helpful for early diagnosis of IVLBCL [8].

CNS vasculitis is characterized histologically by inflamed cerebral vessel walls and is a heterogeneous group of primary and secondary disorders. A diagnosis of primary angiitis of the CNS is a diagnosis of exclusion and other etiologies will need to be considered first. These etiologies include systemic vasculitis (Wegener's granulomatosis, polyarteritis nodosa, and eosinophilic granulomatosis with polyangiitis), infections such as tuberculosis and aspergillus, intravascular lymphoma, which can be intraluminal or infiltrating the vessel walls, cerebral amyloid characterized by heavy beta-amyloid deposition, and substance abuse such as cocaine or amphetamine [14].

In conclusion, with the increased recognition of renal involvement of IVLBCL, a high index of suspicion of this disease entity is required. This patient's diagnosis of IVLBCL was established while he was on rituximab and corticosteroids to treat his primary angiitis of the CNS, which was likely the CNS manifestation of his lymphoma rather than a separate primary vasculitis disease. Despite thorough workup with brain imaging, lumbar puncture, and brain biopsy, this diagnosis of IVLBCL with renal and CNS involvement was only made with a kidney biopsy and the pathology findings were unusual, showing negative CD20 in the atypical lymphoid cells after treatment with rituximab. The case highlights the extranodal manifestations and diffuse nature of this disease. Early recognition is critical as treatment with rituximab-based therapies, such as R-CHOP, intrathecal chemotherapy, and high-dose methotrexate, has significantly improved survival rates, though relapses remain common.

## Data Availability

The clinical and laboratory data used to support the findings of this study are included within the article.
